# Involvement of hippocampal acetylcholinergic receptors in electroacupuncture analgesia in neuropathic pain rats

**DOI:** 10.1186/s12993-016-0096-x

**Published:** 2016-04-12

**Authors:** Shu Ping Chen, Yu Kan, Jian Liang Zhang, Jun Ying Wang, Yong Hui Gao, Li Na Qiao, Xiu Mei Feng, Ya Xia Yan, Jun Ling Liu

**Affiliations:** Department of Physiology, Institute of Acupuncture and Moxibustion, China Academy of Chinese Medical Sciences, 16 Nanxiaojie Street, Dongzhimennei, Beijing, 100700 China; Department of Biochemistry and Molecular Biology, Institute of Acu-Moxibustion, China Academy of Chinese Medical Sciences, Beijing, China

**Keywords:** Neuropathic pain, Electroacupuncture analgesia, Stimulating frequencies, Hippocampus, M1 muscarinic acetylcholinergic receptor (M1 mAChR), M2 mAChR, Alpha-4 nicotinic AChR (nAChR), Beta-2 nAChR

## Abstract

**Background:**

Cumulating evidence has shown a close correlation between electroacupuncture stimulation (EAS) frequency-specific analgesic effect and central opioid peptides. However, the actions of hippocampal acetylcholinergic receptors have not been determined. This study aims to observe the effect of different frequencies of EAS on the expression of hippocampal muscarinic and nicotinic acetylcholinergic receptors (mAChRs, nAChRs) in neuropathic pain rats for revealing their relationship.

**Methods:**

Forty male Wistar rats were randomly and equally divided into sham, CCI model, 2, 2/15 and 100 HzEA groups. The neuropathic pain model was established by ligature of the left sciatic nerve to induce chronic constriction injury (CCI). EAS was applied to bilateral Zusanli (ST36) and Yanglingquan (GB34) for 30 min, once daily for 14 days except weekends. The mechanical pain thresholds (withdrawal latencies, PWLs) of bilateral hindpaws were measured. The expression levels of hippocampal M1 and M2 mAChR, and α4 and β2 nAChR genes and proteins were detected by quantitative RT-PCR and Western blot, separately. The involvement of mAChR and nAChR in the analgesic effect of EAS was confirmed by intra-hippocampal microinjection of M_1_mAChR antagonist (Pirenzepine) and α4β2 nAChR antagonist (dihydro-beta-erythroidine) respectively.

**Results:**

Following EAS, the CCI-induced increase of difference values of bilateral PWLs on day 6 and 14 was significantly reduced (P < 0.05), with 2/15 Hz being greater than 100 Hz EAS on day 14 (P < 0.05). After 2 weeks’ EAS, the decreased expression levels of M1 mAChR mRNA of both 2 and 2/15 Hz groups and M1 mAChR protein of the three EAS groups, α4 AChR mRNA of the 2/15 Hz group and β2 nAChR protein of the three EAS groups were considerably increased (P < 0.05), suggesting an involvement of M1 mAChR and β2 nAChR proteins in EAS-induced pain relief. No significant changes were found in the expression of M2 mAChR mRNA and protein, α4 nAChR protein and β2 nAChR mRNA after CCI and EAS (P > 0.05). The analgesic effect of EAS was abolished by intra-hippocampal microinjection of M_1_mAChR and α4β2 nAChR antagonists respectively.

**Conclusions:**

EAS of ST36-GB34 produces a cumulative analgesic effect in neuropathic pain rats, which is frequency-dependent and probably mediated by hippocampal M1 mAChR and β2 nAChR proteins.

## Background

It has been well documented that chronic pain including neuropathic pain involves complex brain circuits for sensory, emotional, cognitive and interoceptive processing [1, 2]. The hippocampus, one of the limbic structures for antinociception [3], has been shown to undergo significant changes including reduction of hippocampal volume, learning and emotional deficits, sustained endocrine stress response, etc., in chronic pain syndrome patients [4–8], and abnormal expression of cytokine, extracellular signal-regulated kinase, neurokinin-1 (NK-1) receptor, etc., in experimental chronic neuropathic pain animals [9–11].

Among the neurotransmitters or mediators involving the chronic pain induced abnormal behavioral deficits, acetylcholine (ACh) is an important candidate in the hippocampus. Studies repeatedly demonstrated that cholinergic compounds produced antinociceptive effects in the rhesus monkey [12], cat [13] and rat [14–17]. Systemic administration of cholinesterase inhibitors which cross the blood brain barrier was found to produce analgesia and enhance analgesia from opiates [18, 19].

Early studies on acupuncture analgesia have already shown that hippocampal cholinergic activities are involved in acupuncture analgesia [20–22]. But, related researches are relatively fewer. In recent years, we demonstrated that both hippocampal and hypothalamic cholinergic activities were involved in the cumulative analgesia induced by repeated electroacupuncture stimulation (EAS) of “Zusanli” (ST36) and “Yanglingquan” (GB34) in rats with chronic constrictive injury (CCI) of the sciatic nerve [23, 24]. However, the detailed mechanisms underlying involvement of ACh in analgesia are still not clear.

Moreover, stimulating parameters, particularly the frequency, are important factors affecting the analgesic effect of EAS [25]. Chen and Wang [26] reported that in 252 cases of soft tissue injury-induced pain patients, 100 Hz EAS was significantly better than 2 Hz EAS in the cure rate and effective rate for pain. On the contrary, Zou et al. [27] observed that in 90 cases of acute arthritis patients, 2 Hz EAS was apparently better than 100 Hz EAS in pain-relief. Experimental studies also showed contradictory results about the analgesic effect of different frequencies of EAS in different acute pain models [28, 29].

In regard to the analgesic mechanisms of different EAS frequencies, majority of researches focused on the release of endogenous opioid peptides, one of which is Han’s and his colleagues’ well-known conclusion that 2 Hz EAS induced analgesia mediated by the release of met-enkephalin (M-ENK) and β-endorphin (β-EP), while 100 Hz EAS via dynorphin-A (DYN-A) in the central nervous system [30–34]. Latter, 5-HT in the brainstem [35, 36], catecholamine [37, 38], hypothalamic substance P [39], cholecystokinin (CCK) and CCK-A and -B receptors [40] were found to be involved in the frequency-specific analgesic effect. However, to our knowledge, there have been no any reports on the cholinergic involvement of frequency-specific analgesic effect of EAS. For this reason, the present study was designed to observe the effect of EAS at different frequencies on pain behaviors and expressions of hippocampal muscarinic acetylcholine receptor (mAChR) and nicotinic acetylcholine receptor (nAChR) in CCI-induced neuropathic pain rats, thereby, to better our understanding on the mechanism of acupuncture in the management of neuropathic pain.

## Methods

### Animals and grouping

Male Wistar rats (230–270 g) were obtained from the Experimental Animal Center of Peking Union Medical College (Beijing, China), and housed within the animal care facilities in the Institute of Acupuncture and Moxibustion, China Academy of Chinese Medical Sciences. Rats were housed in a climate-controlled room on a 12 h light/dark cycle with food and water provided ad libitum. Animals were randomly divided into control (sham ligature), CCI model, CCI + 2 HzEA, CCI + 2/15 HzEA and CCI + 100 HzEA groups (n = 8 in each group). For verifying the effect of hippocampal mAChRs and nAChRs on EA analgesia, additional 30 male Wistar rats were randomized into control, model, saline-injection (saline), M1R-antagonist and nAChR-antagonist groups (n = 4 in each group). All experimental procedures were approved by the Institute of Acupuncture and Moxibustion of China Academy of Chinese Medical Sciences, and performed according to the “Guidelines for Laboratory Animal Care and Use” of the Chinese Ministry of Science and Technology (2006).

### Neuropathic pain model and pain threshold detection

Following a 7-day environmental adaptation, rats were anesthetized (25 % urethane plus 1.5 % chloralose, 0.4 mL/100 g body weight) and received CCI of the sciatic nerve as previously described [41]. Briefly, the left posterolateral thigh was routinely sterilized, and a 2 cm incision was made through the skin. The left common sciatic nerve was exposed at mid-thigh by blunt dissection through the biceps femoris. Four constrictive ligatures (4–0 silk sutures) were tied around the nerve at the distal end close to the bifurcation site (about 1 mm space between every two ligatures). The ligature was alright until a moderate muscular contraction of the leg was seen. The same procedure was performed for rats in the control group but without nerve ligature. The incision was then closed using 5–0 silk sutures.

The paw withdrawal latency (PWL) (i.e., the mechanical pain threshold) of the bilateral hind paws was detected using a Dynamic Plantar Aesthesiometer (Ugo Basile, 37450, Italy) before CCI, 3 days after CCI, and 1, 6 and 14 days after EA treatment. Rats were placed on a metal mesh table and in an individual plexiglass housing at the same time. The steel rod (0.5 mm diameter) of 37450 was pushed up to the plantar surface of the hind paw with increasing force (2.5 g/s). The cutoff pressure was set to be 30 g and the threshold was recorded when the rat retracted its foot abruptly responding to the increased pressure. Thermal pain threshold was detected using PLANTAR TEST (Ugo Basile, 37370, Italy). The radiant heat source was focused on the plantar surface of the hindpaw, and light intensity was preset to obtain a baseline latency of approximately 15 s. Each rat underwent three trials with a 5-min inter-trial interval, and the mean value of these trials was used as the PWL. To minimize differences in individual animals, the difference value of PWL (PWLD) between the healthy and the affected hindpaws was calculated. Their hypersensitivities were defined as the presence of at least a 20 % decrease in pain threshold compared with pre-CCI baselines. Rats not exhibiting pain hypersensitivity after CCI were discarded.

### Electroacupuncture intervention

Bilateral Zusanli (ST 36, 5 mm beneath the capitulum fibulae and lateral posterior to the knee-joint) and Yanglingquan (GB 34, about 5 mm superior-lateral to ST 36) were punctured with filiform needles (Gauge 28), respectively, and electrically stimulated using a HANS EA Apparatus (LH202, Beijing Huawei Industrial Developing Company, Beijing, China). EA (2 Hz, alternative 2/15, 100 Hz, 1 mA) was administered for 30 min, once per day for 1 or 2 weeks beginning from day 4 after surgery. For rats of hippocampal injection of M1mAChR and α4β2 nAChR antagonists, EA (2/15 Hz, 1 mA) was administered for 30 min, once daily for 5 days before the injection.

### Intra-hippocampal injection

Under anesthesia with chloral hydrate (400 mg/kg i.p),the rat who experienced 3–5 days recovery from CCI operation was fixed in a stereotaxic instrument (Stoelting Co, USA) and stainless steel 26-gauge cannulae were implanted into the bilateral dorsal hippocampus (anteroposterior, −3.6 mm; medial-lateral, ± 3.1 mm; dorsoventral, −2.4 mm) according to Paxinos’ and Watson’s Atlas [The Rat Brain in Stereotaxic Coordinates, 6th edition from George Paxinos, Charles Watson], and fixed with dental cement. The stainless steel obdurator was remained in the cannulae before injection for preventing obstruction. After implantation of the cannula, each rat was allowed to have a recovery period of at least 7 days before the experiments. For hippocampal injection, a mini-size pump (KDS310 Plus, kdScientific, USA) connected to the catheter was used for continuous infusion of antagonists [pirenzepine hydrochloride, M1mAChR selective antagonist, Sigma; dihydro-beta-erythroidine, an α4 β2 nAChR antagonist; Tocris, UK; dissolved in sterile saline to a concentration of 10 nmol/μL] or normal saline at a rate of 1 μL/h/hemisphere. The injector was remained connected for an additional 1 min to allow the drug diffusion away from the tip of the cannula. Before and 3−5 days after CCI surgery, 1, 3, and 5 days after hippocampal injections and EAS (2/15 Hz, 1 mA duration of 30 min), the thermal and mechanical pain thresholds were detected respectively. Rats with cannula-desquamation or death were excluded in the present study. The location of the intra-hippocampal catheter was confirmed by pantamine sky blue (0.2 %, 1 μL) microinjection after completion of the experiments.

### Quantitative RT-PCR analysis

At the end of EA treatments, 6 randomly-selected rats of each group were deeply anesthetized with the anesthetics mentioned above, and the right hippocampus tissue was separated. Total RNA was extracted from the tissue using Trizol reagent (Invitrogen, USA). First-strand cDNA was synthesized by a reverse transcriptase kit (Invitrogen, USA) according to the manufacturer’s instructions, and used as the template for quantitative RT-PCR analysis on a ABI 7500 fast real time system (Applied Biosystems, CA, USA), with β-actin as an internal control. Each reaction included 2 μl (25 ng/μl) of cDNA and was performed in triplicate.The primer sequences were as follows.

β-actin (NM_031144.3): 5′-GGAGATTACTGCCCTGGCTCCTA-3′ (Forward), 5′-GACTCATCGTACTCCTGCTTGCTG-3′ (Reverse) (bp:150); M1 mAChR (NM_080773.1): 5′-GCTGGAAGGAAGAAGAAGAGGAGGA-3′ (Forward), 5′-GCTGGAAGGAAGAAGAAGAGGAGGA (Reverse) (bp:160); M2 mAChR (NM_031061): 5′-CCATTCTCTTCTGGCAGTTCATCGT-3′ (Forward), TCTTTATTCTACTCTTGCTTGCCCG (Reverse) (bp: 183); β2 nAChR (NM_019297.1): 5′-CGGGAAGCAGTGGATGGCGTA -3′ (Forward), 5′-GTCCTCCCTCACACTCTGGTCATCA-3′ (Reverse) (bp: 78); α4 nAChR (NM_024354.1): 5′-ATGGATGAAACCTACCTGATGAGCA-3′ (Forward), 5′-GCTGGGGGTTGTAGCAGGCAC-3′ (Reverse) (bp: 130). Cycling conditions were as follows: denaturation (95 °C for 10 min), amplification and quantitation (95 °C for 15 s, 60 °C for 60 s) repeated 40 times, and 72 °C for 32 s, with a single fluorescence measurement at the end of 72 °C for 32 s segment) repeated 35 times, a melting curve program (60–95 °C with a heating rate of 0.1 °C/s and continuous fluorescence measurement) and a cooling step to 40 °C. Quantitative RT-PCR data were normalized with β-actin mRNA levels. Relative mRNA levels were expressed as 2-∆∆Ct values.

### Western blot analysis

Fresh contralateral hippocampal tissues were initially homogenized in lysis buffer containing a cocktail of phosphatase and proteinase inhibitors (Roche). Tissue protein concentrations were determined using the BCA protein assay kit (Pierce, Rockford). Protein samples(total 40 μg, 20 μl) were electrophoretically separated on a SDS-PAGE gel and transferred to polyvinylidene difluoride membranes (0.45 um pores; Millipore, Bedford, MA). The membranes were blocked with 2 % bovine serum albumin (BSA, Amresco, USA) solution for 2 h at room temperature (RT) and then incubated overnight at 4 °C with rabbit anti-M1 mAChR (1:2000, SC-9106, Santa) and mouse anti-M2 mAChR (1:2000, ab2805, Abcam), rabbit-anti- α-4nAChR (1:5000, Abcam, ab124832), rat anti-β2 nAChR (1:4000, ab24698, Abcam) primary antibodies. All antibodies were diluted in Tris-buffered saline solution containing 0.5 % Tween 20 (TBST) and 3.0 % BSA-TBSA. After washing in TBST, the blots were incubated with horseradish peroxidase (HRP)-conjugated secondary antibody for 2 h at RT (1:20,000; goat anti-rabbit immunoglobulin G; and 1:10,000: goat anti-mice IgG, 1:5000: goat anti-rat IgG). Following the rinse in TBST, the blots were developed using enhanced chemilluminescence for 1 min and exposed onto chemiluminescent films. For densitomentric analyses, the blots were scanned and quantified using TotalLab Quant analysis software (Totallab Limited, England), and the result was expressed as the ratio of target gene immunoreactivity to GAPDH immunoreactivity.

### Statistical analysis

Data were expressed as mean ± standard deviation (mean ± SD). Data were analyzed via one-way ANOVA (for mRNA and protein expression) or two-way ANOVA (for pain thresholds) when appropriate, followed by least significant difference (LSD) tests for comparing data between groups. A value of P < 0.05 was considered statistically significant.

## Results

### Effect of EAS on pain threshold

**Fig. 1:**
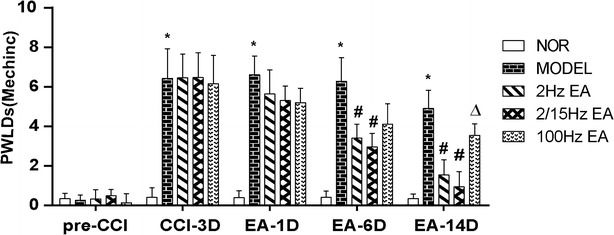
Effect of different frequencies of electroacupuncture stimulation (EAS) of Zusanli (ST36) and Yanglingquan (GB34) on difference values of the bilateral hindpaw withdrawal latencies (PWLDs, mechanical pain threshold) in neuropathic pain rats (mean ± SD, g, n = 8 in each group); *NOR* normal group, Model group: CCI (chronic compressive injury), CCI + 2 Hz EAS group: 2 Hz EA, CCI + 2/15 Hz EAS group: 2/15 Hz EA, CCI + 100 Hz EAS group: 100 Hz EA (the same in Figs. 2, 3); *P < 0.05, vs the normal group; ^#^P < 0.05, vs the model group; ^∆^P < 0.05, vs the 100 Hz EAS group

As showed in Fig. 1 that after ligature of the sciatic nerve to induce CCI, the PWLDs were significantly increased in rats of the model group (P < 0.05, Fig. 1), suggesting a mechanical hypersensitivity 3 days after surgery. In comparison with the CCI model group, the PWLDs were pronouncedly decreased in rats of the CCI + EA2 Hz group and CCI + EA2/15 Hz group on day 6 and day 14 after CCI-operation (P < 0.05), and 2 Hz and 2/15 Hz EAS were notably superior to that of 100 Hz EAS in reducing PWLDS (P < 0.05, Fig. 1). In addition, the analgesic effect was gradually increased along with the extension of EA intervention, suggesting an accumulative effect of EA treatments.

### Effect of EAS on hippocampal M1 and M2 mAChR gene and protein expression

#### M1 mAChR

**Fig. 2:**
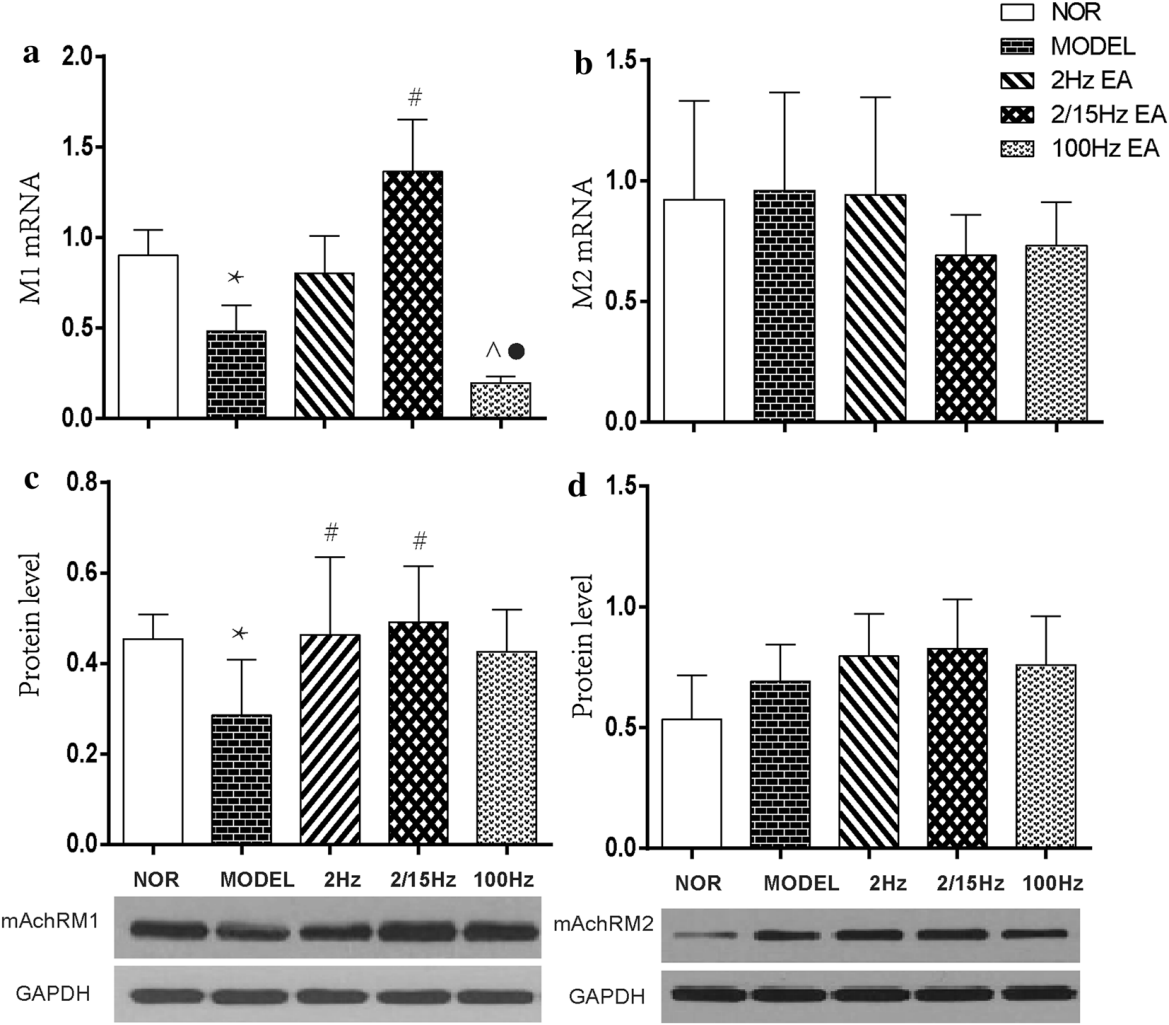
Effect of different frequencies of EAS of ST36-GB34 on expression of M1 and M2 AChR mRNA and proteins in the hippocampus in neuropathic pain rats (mean ± SD, n = 6 in each group); **a** M1 AChR mRNA, **b** M1 AChR protein; **c** M2 AChR mRNA, **d** M2 AChR protein; *P < 0.05, vs the normal group; ^#^P < 0.05, vs the model group; ^P < 0.05, vs the 2 Hz EAS group; $^\bullet$P < 0.05, vs the 2/15 Hz EAS group

In comparison with the control group, the expression levels of both M1 mAChR mRNA and protein in the hippocampus were significantly down-regulated (P < 0.05, Fig. 2a), suggesting an involvement of M1 mAChR in the nociceptive reactions following CCI. When compared with the CCI group, the expression levels of M1 mAChR mRNA in both CCI + EA2 Hz and CCI + EA2/15 Hz groups and protein expression in CCI + EA2 Hz, CCI + EA2/15 Hz and CCI + EA100 Hz groups were considerably upregulated following 2 weeks’ treatment (P < 0.05, Fig. 2b). The expression levels of M1 mAChR mRNA in both CCI + EA2 Hz group and CCI + EA2/15 Hz group were remarkably higher than that in the CCI + EA100 Hz group (P < 0.05, Fig. 2a). No significant differences were found among the three EAS groups in M1 mAChR protein expression levels, and between the CCI + EA2 Hz and CCI + EA2/15 Hz groups and between the CCI group and CCI + EA100 Hz groups in M1 mAChR mRNA expression levels (P > 0.05).

#### M2 mAChR

Compared to the control group, there were no apparent changes in the expression levels of both M2 mAChR mRNA and protein in the hippocampus after CCI surgery (P > 0.05, Fig. 2c, d). In comparison to the CCI group, no obvious changes were found in the expression levels of both M2 mAChR mRNA and protein after EAS (P > 0.05, Fig. 2c, d).

### Effect of EAS on hippocampal α4 and β2 nAChR gene and protein expression

#### α4 nAChR

**Fig. 3:**
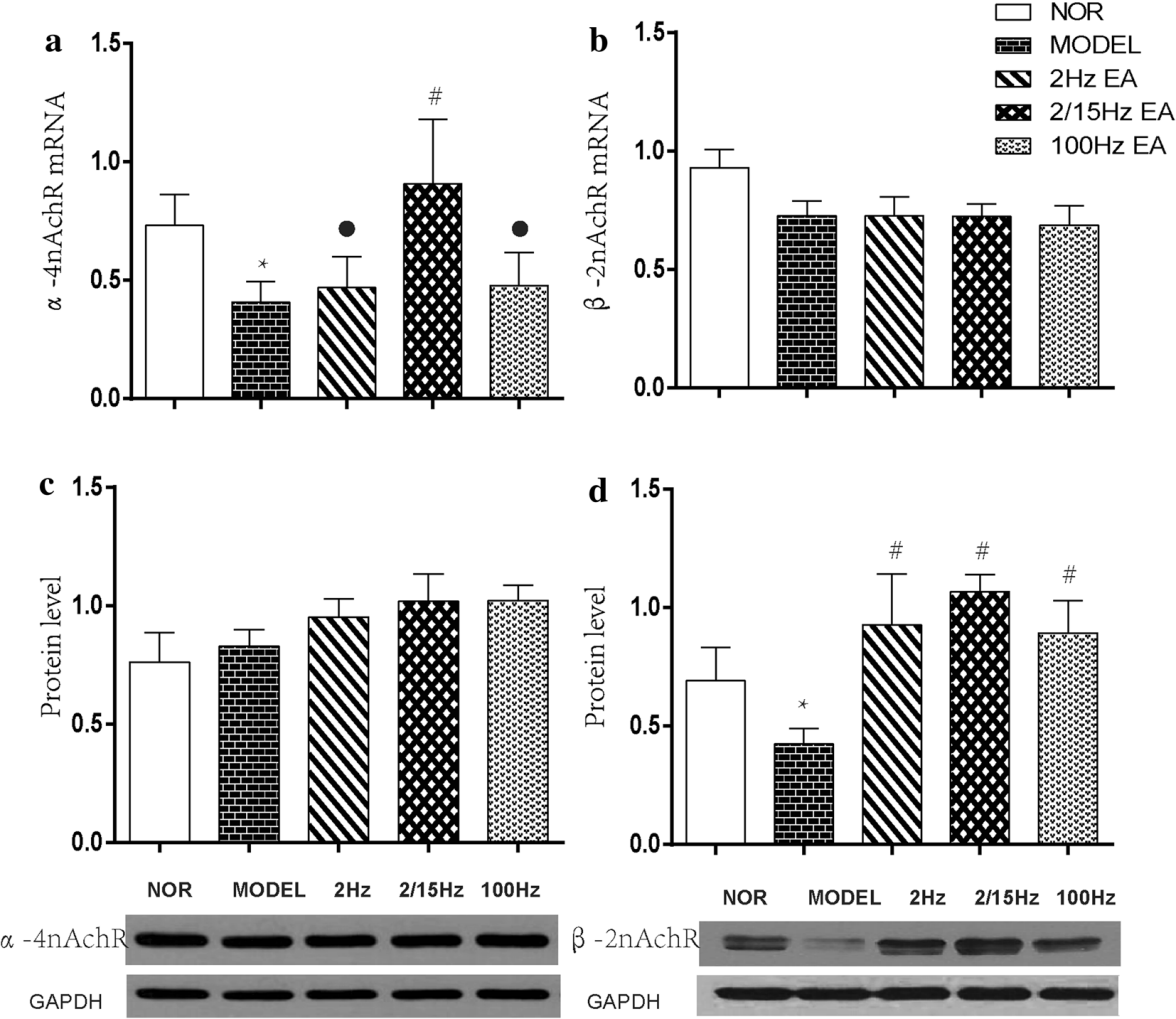
Effect of different frequencies of EAS of ST36-GB34 on expression of α4 nAChR and β2 nAChR mRNA and proteins in the hippocampus in neuropathic pain rats (mean ± SD, n = 6 in each group); **a** α4 nAChR mRNA, **b** α4 nAChR protein; **c** β2 nAChR mRNA, **d** β2 nAChR protein; *P < 0.05, vs the normal group; ^#^P < 0.05, vs the model group; $^\bullet$P < 0.05, vs the 2/15 Hz EAS group

Quantitative real-time PCR detection of both α4 nAChR and β2 nAChR mRNA showed that only α4 nAChR mRNA expression in the hippocampus was significantly down-regulated after CCI in the CCI group (P < 0.05, Fig. 3a), while β2 nAChR mRNA expression had no marked changes in the CCI and the three EAS groups (P > 0.05, Fig. 3c). Following EAS, α4 nAChR mRNA expression level was obviously up-regulated in the CCI + EA2/15 Hz group (P < 0.05, Fig. 3a), not in the CCI + 2 Hz and CCI + 100 Hz groups (P > 0.05).

#### β2 nAChR

Western blot detection displayed that hippocampal α4 nAChRprotein expression had no apparent changes after CCI in the CCI and the three EAS groups (P > 0.05, Fig. 3b), while β2 nAChR protein expression was significantly down-regulated in the CCI group (P < 0.05, Fig. 3d). After EAS, β2 nAChR protein expression levels of the CCI + EA2 Hz, CCI + EA2/15 Hz and CCI + EA100 Hz groups were considerably upregulated (P < 0.05, Fig. 3d). No significant differences were found among the three EAS groups in the expression of β2 nAChR protein (P > 0.05).

### Effect of hippocampal injection of M1mAChR and α4β2 nAChR antagonist on EA analgesia

**Fig. 4:**
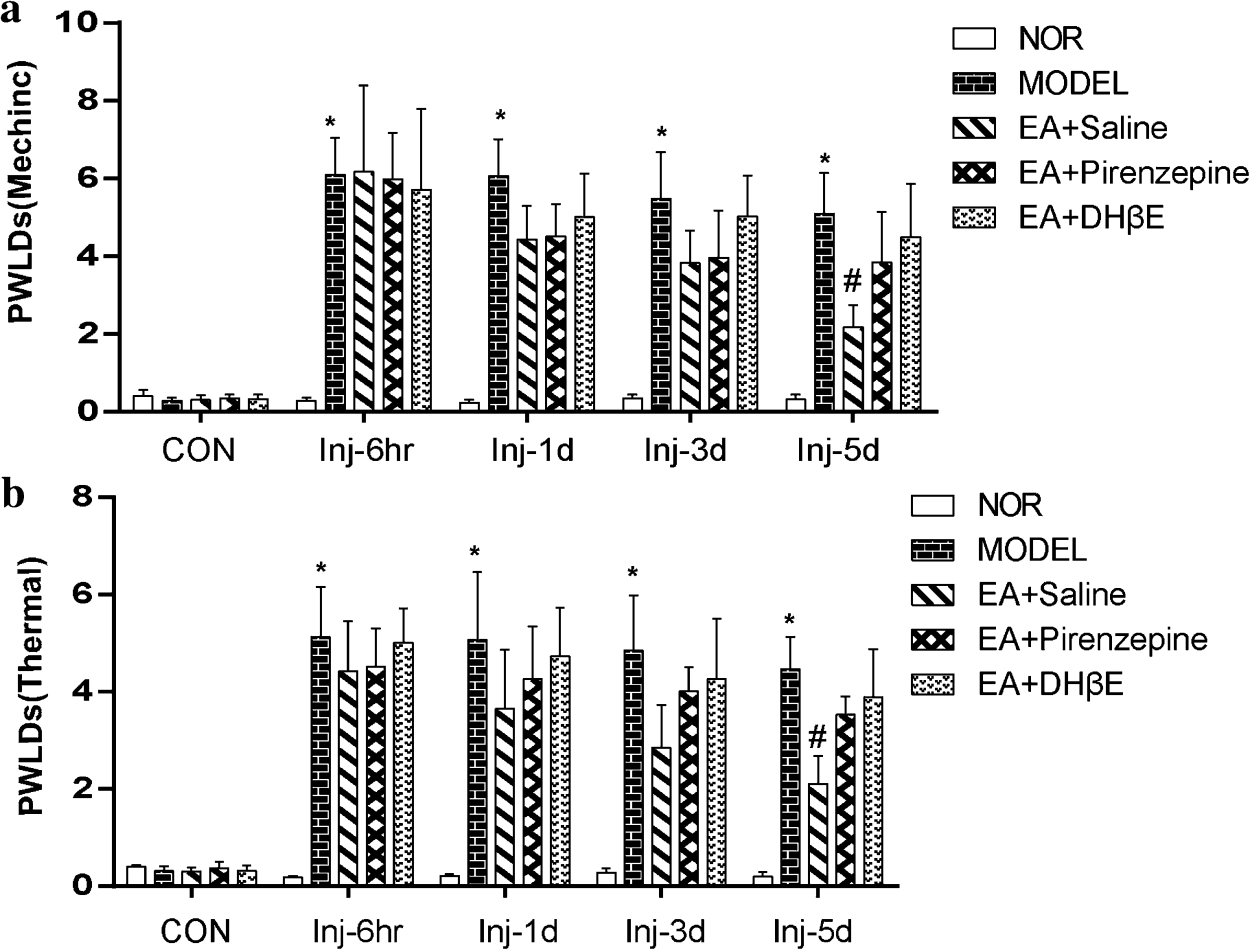
Effect of intra-hippocampal injection of M1mAChR selective antagonist (pirenzepine hydrochloride) and α4β2 nAChR antagonist (Dihydro-beta-erythroidine, DHβE) on pain threshold (**a** mechanic, g; **b** thermal, sec) in neuropathic pain rats (mean ± SD, n = 4 in each group); *P < 0.05, vs the normal group; ^#^P < 0.05, vs the model group

Results of Fig. 4 showed that the PWLDs of the model group at time-points of 6 h, 1, 3 and 5 days after CCI were significantly increased in the model group (P < 0.001). Compared to the model group, the PWLD was significantly decreased in the EA + saline group (P < 0.05) but not in the EA + Pirenzepine and EA + DHβE groups on day 5 after EAS (P > 0.05), suggesting a reduction of EA analgesia after intra-hippocampal injection of M1mAChR and α4β2 nAChR antagonists.

## Discussion

Findings of the present study revealed that after one and two weeks’ EAS at 2 Hz and alternative frequencies of 2/15 Hz but not 100 Hz, the mechanical pain thresholds were significantly increased, and the effects of 2 and 2/15 Hz were superior to that of 100 Hz EAS beginning on day 6 and significantly on day 14 after EAS, meaning a better analgesic effect of lower frequency EAS in neuropathic pain rats. These results are basically identical to Zou’s and colleagues’ outcomes acquired in acute arthritis patients [27], Romita’s and colleagues’ study [42] and Li’s [16], Mayor’s [43], and Wang’s [29] reviews about experimental studies, but different to Chen’s and Wang’s outcomes obtained in soft tissue injury patients which 100 Hz EAS was better than 2 Hz in pain relief [26] and also different to Hahm’s [28] and Chang’s and colleagues’ [44] results in which a comparable pain relief of 2 Hz and 100 Hz EAS was observed in ankle sprain rats and inflammatory pain mice.

Results of real-time RT-PCR and WB of the present study showed that following 2 weeks’ EAS, both 2 and 2/15 Hz could obviously reverse CCI-induced decrease of M1mAChR mRNA and protein expression, and so did 100 Hz EAS in upregulating M1mAChR protein expression. The effects of 2/15 Hz EAS were notably better than those of 100 Hz EAS in upregulating M1 mAChR mRNA and α4 nAChR mRNA expression, displaying a closer correlation between the EA analgesia at 2/15 Hz and M1 mAChR and β2 nAChR protein expression levels in CCI rats, rather than M2 mAChR protein expression. Following intra-hippocampal microinjection of M1 mAChR antagonist Pirenzepine and α-4β-2nAChR antagonist DHβE, both the increased thermal pain and mechanical pain thresholds were suppressed, denoting an involvement of hippocampal M1 mAChR and α-4β2 nAChR in mediating the cumulative analgesic effect of EAS.

It is well known that the gene expression contains both transcription and transduction, and changes of the target genes detected by PCR only reflects up- or down-regulation of a molecule at the transcription level with no relevance to physiological functions, while the relative expression of proteins detected by WB is simply referred to protein transduction, and directly involves functional activities. Thus, it is understandable that the expression levels of genes and proteins of α4 nAChR and β2 nAChR did not show a positive correlation. No selective antagonists for simple α4 nAChR and simple β2 nAChR were found, we were forced to observe the effect of α4β2 nAChR antagonist on EA analgesia.

Studies using retrograde tracing and excitotoxin lesions [45], ChAT and/or AChE pharmacohistochemical regimen [46] and co-cultured slices of septum and hippocampus (not single cultures of hippocampus) in combination with immunocytochemistry for choline acetyltransferase (AChT) [47] demonstrated that the hippocampal formation is innervated primarily by cholinergic neurons located in the vertical limb of the diagonal band and in the medial septum. It also has been shown that the cholinergic, opioidergic and GABAergic systems of the hippocampus were involved in the modulation of antinociception, and the cholinergic transmission may activate the release of endorphins/enkephalin from interneurons of the dorsal hippocampus to inhibit GABAergic neurons, resulting in antinociception [48].

The hippocampus expresses a broad range of mAChRs, with the M1 and M3 receptors being mainly expressed on principal neurons and M2 and M4 receptors on interneurons [49, 50]. However, only fewer studies have shown roles of different subunits of hippocampal mAChRs and nAChR in pain modulation. For instance, intra-CA3 microinjection of ACh or ACh agonist pilocarpine and mAChRs antagonist atropine showed that hippocampal mAChRs were complicated in the modulation of the nociceptive response by modulating the electrical activities of pain-excited or -inhibited neurons in the hippocampal CA1 and CA3 regions of normal rats experiencing electrical stimulation of the ischial nerve [16, 51]. The hippocampal M1 mAChR was shown to be involved in moderate pain reactions in repeated intraperitoneal injection-induced moderate pain in C57BL/6J mice [52].

The septo-hippocampal pathway was also thought to activate nAChRs, because intraperitoneal injection of nAChR antagonist chlorisondamine induced an antinociceptive effect in acute thermal (hot box) and persistent chemical (formalin test) pain rats [53]. At least three distinct functional nAChRsubtypes (α7, α4 β2, α3 β4) could be detected in the hippocampal region [54], and most of the rat hippocampal heteromeric nAChRs contain α4 and β2 subunits, with the 3H] epibatidine –labeled α4β2 and α4β2α5 subtypes accounting for about 40 and 35 %, respectively [55]. In accordance with Mitsui’s report [56], in spite of no obvious physical or neurological deficit in AChR knockout mice, pharmacological, biochemical, electrophysiological, neuroanatomical and behavioral analyses revealed that these AChR subunits may form a component of the nicotinic pain pathways modulating the antinociceptive effect of nicotine. Experimental results of tail-flick and hot-plate tests indicated that α4 β2 nAChRs were important in mediating neuronal nicotinic analgesia in both spinal and supraspinal responses in knockin mice expressing hypersensitive α4 β2 nicotinic receptors [57]. However, there still have been no any reports on gene and protein expression of hippocampal nicotinic receptors involving pain modulation up to now.

EAS frequency is considered to be an important parameter affecting its analgesic effect. Up to now, many studies focus on the low frequency 2–5 Hz, medium frequency 15–40 Hz, and high frequency 100–200 Hz for various pain models, which were chosen in consideration of nervous tissue responses. If the frequency is over 100 Hz, the reactions of the nerve tissue may not truthfully follow the electrical stimulation [25].

Using rat tail-flick tests, Silva et al. [58] observed that the analgesic effect of 2 Hz EAS of ST36 and Sanyinjiao (SP6) lasted longer than that of 100 Hz EAS. Intrathecal administration of antagonists of α1- (WB4101) and α2- (idazoxan) adrenoceptors and serotonergic (methysergide), opioid (naloxone), muscarinic (atropine), GABA (A) (bicuculline) and GABA (B) (phaclofen) receptors showed that the analgesic effect of 2 HZ EAS was inhibited by naloxone or atropine, being less intense and shorter after α1 or α2 inhibition, and lasting shorter after 5-HT, GABAA, or GABAB receptor suppression; while that of the 100 Hz EAS was less intense and shorter after opioid and muscarinic suppression, being less intense and longer after GABAB inhibition, shorter after 5-HT or GABAA inhibition, and remained unchanged after α1 or α2 inhibition. It suggests that the analgesic efficacy (intensity) of 2 Hz EAS depends on noradrenergic descending inhibition and involves spinal opioid and muscarinic mechanisms, whereas the duration of the analgesic effect relies on both noradrenergic and serotonergic descending control, and involves spinal GABAergic regulation. On the contrary, the analgesic efficacy of 100 Hz EAS involves spinal muscarinic, opioid, and GABAB activation, while the duration of the effects is affected by spinal serotonergic, muscarinic, opioid, and GABAA activation. Their further study [59] demonstrated that the cholinergic muscarinic, μ-opioid, GABAA and 5-HT1 mechanisms in the dorsal -anterior pretectal nucleus (APtN) and μ-opioid and 5-HT1 mechanisms in the ventral APtN were involved in 2 Hz EAS analgesia, while the μ-opioid and 5-HT1 mechanisms in the vAPtN but not in the dAPtN were complicated in 100 Hz EAS analgesia.

Recently, using cDNA microarray, Wang et al. [60] demonstrated in the rat that more genes were differentially regulated by 2 Hz EA than 100 Hz EA of ST36 and SP6 (154 vs. 66 regulated genes/ESTs) in the arcuate nucleus (Arc) region, especially those related to neurogenesis. Results of fMRI in combination with behavior tests showed that following 2 and 100 Hz EAS in the human body, the regional cerebral blood flow (CBF) signals revealed a trend of early activation with later inhibition; and a positive correlation between analgesia and the regional CBF change was observed in the anterior insula in the early stage, whereas a negative relationship was found in the parahippocampal gyrus in the later stage. TEAS analgesia was specifically associated with the default mode network and other cortical regions in the 2 Hz TEAS group, ventral striatum and dorsal anterior cingulate cortex in the 100 Hz TEAS group, respectively [61]. Later, it was found in rhesus monkeys that 2 Hz but not 100 Hz TEAS evoked a significant increase in mu-opioid receptor (MOR) binding potential in the anterior cingulate cortex, caudate nucleus, putamen, temporal lobe, somatosensory cortex, and the amygdala which are related to pain and sensory processing [62]. These findings suggest that the mechanisms of low- and high-frequency EAS analgesia are different and partially overlapped.

## Conclusions

Results of our present study showed that in neuropathic pain rats, repeated EA treatment at frequencies of 2 and 2/15 Hz, particularly the later (but not 100 Hz) has a cumulative analgesic effect, which is closely related to their effects in upregulating the expression of hippocampal M1 and β2 nAChR proteins, highlighting the involvement of muscarinic and nicotinic receptor subtypes in EA analgesia for the first time.

## Abbreviations

CCI: chronic constrictive injury
EAS: electroacupuncture stimulation
PWL: paw withdrawal latency
PWLD: difference value of PWL
M1 mAChR: M1 muscarinic acetylcholinergic receptor
M2 mAChR: M2 muscarinic acetylcholinergic receptor,
α-4nAChR: alpha-4 nicotinic acetylcholinergic receptor
Beta-2 nAChR: beta-2 nicotinic acetylcholinergic receptor
